# Five Crucial Challenges in Digital Health

**DOI:** 10.3389/fdgth.2020.536203

**Published:** 2020-12-08

**Authors:** Nicholas Cummins, Björn W. Schuller

**Affiliations:** ^1^Chair of Embedded Intelligence for Health Care and Wellbeing, University of Augsburg, Augsburg, Germany; ^2^The Department of Biostatistics and Health Informatics, Institute of Psychiatry, Psychology and Neuroscience, King's College London, London, United Kingdom; ^3^Department of Computing, Imperial College London, London, United Kingdom

**Keywords:** positioning, overview, survey, grand challenge, digital health

## 1. Introduction

The concept of digital health is not a new or revolutionary one. For example, technologies, such as medical images and telemedicine date back over 100 years (1, 2), while prototype wearable devices have been used to tackle obesity since the 1940s (3). Digital health, however, has had continually transforming effects in an industry that is notoriously resistant to change (4). Moreover, since the mid-1990s when the US National Academy of Medicine began recommending the complete digitization of health data (5), the transforming effects of digital technologies in healthcare has never been more evident. With the advent of wearables and other Internet of Things (IoT) devices, health care is moving further toward personalized and preventative paradigms utilizing ubiquitous technologies which support real-time self-care or monitoring (6).

The ongoing coronavirus disease 2019 (COVID-19) pandemic increases the need for ongoing digital health advancements. For example, traditional face-to-face medical consults increase the risk of infection, emphasizing the need for virtual consultation technologies (7). Similarly, tools are needed to help understand and support the effects of the pandemic on our physical and, in particular, mental health (8). Even without this amplified need, it is clear that digital technologies will continue to transform the healthcare sector again and again. As with Wilhelm Conrad Röntgen—the physicist who discovered X-rays (1)—and many others before, contributions are expected from individuals with technical backgrounds rather than just pure medical. Building on from Kostkova (9), this article highlights a selection of crucial current challenges to overcome to ensure that digital health systems to meet the guiding principle of being *for all anywhere* and at *anytime*.

## 2. Societal Factors

While technology is at the heart of any digital health system, the related transformations cannot be viewed purely through a technological lens (10). Digital technologies need to deliver affordable, easy-to-use healthcare solutions to a growing and aging population in which new technologies are often slow to be adopted and accepted by the general populace (11). Factors that influence this lack of acceptance include regulatory factors, such as uncertainties surrounding digital health policies and legislation as well as a perceived lack of accountability within the commercial sector. The commercial sector itself has challenges related to the complexity of the multinational nature of the digital health market and the need to operate within the constraints of a multitude of different health systems. Low levels of digital and health literacy—and thus digital health literacy—in the general population, especially in the elderly, is also a major contributing factor.

Substantial efforts, therefore, needed to be made to normalize the use of digital health at a societal level. These efforts should include upskilling both health professional and the general public through broad-reaching educational initiatives (11). Processes are also needed to assess the suitability of digital health solutions from the perspective of all stakeholders, especially those representing the patient and clinician end-users groups (12). To ensure wider adaption, the next generation of digital technologies should support and engage users in such a way that fosters equality and inclusivity, resulting in improved healthcare solutions for all (13, 14).

COVID-19 has highlighted the need to adapt and update clinical care delivery systems (15). Concerns have been raised that efforts to move away from traditional face-to-face medicine and toward remote, digital, solutions have highlighted, such as the already existing socioeconomic gaps between groups of people who can easily access and use such services and those who cannot (16, 17). Social media, while playing a vital role in supporting communication between social and family groups during lockdowns, has also made it considerably easier to spread medical misinformation across societies (18, 19). Societal and governmental efforts are urgently needed to help counter this negative phenomenon (19). Contact tracing using remote apps has enabled mass data collection to aid public health and research efforts (16). However, this raises concerns relating to data ownership and other ethical concerns (16, 20).

## 3. Ethical Challenges

The increasing digitization of healthcare and the growth of mobile and IoT devices as data collection tools raises many ethical issues. One commonly recurring theme relates to the exact nature of the role of consumer tech companies, such as Amazon, Apple, Google, Facebook, or Samsung, who have all entered the digital health domain (21). In particular, such companies offer solutions for collecting, storing and analyzing health data which raises issues relating to privacy, data protection and informed consent (21–23). The nature of health data is also changing; we are now collecting more private user-generated data, particularly data harvested from social media and through wearable technologies, than ever before.

As well as the issues mentioned above of privacy, protection, and consent, ethical concerns relating to data ownership are frequently discussed in the literature [e.g., (23, 24)]. The growth of apps and technologies developed for a consumer market blurs the lines between what is medical and non-medical devices and raises ethical challenges relating to how to regularize such technologies (22, 25). This issue is exacerbated by the speed of advancements and increasing globalization of healthcare solutions (25, 26).

## 4. Increased Connected Health Solutions

A core aim of digital health is to help facilitate the circulation of data between patients, devices, and clinicians (27). Increasing this connectivity enables smarter and more timely sharing of information between clinicians and patients and has strong links to the *predictive, preemptive*, and *personalized* principles of digital health (28). Connected health solutions are also a key element in response to the COVID-19 pandemic (29). However, increased connected health solutions come with increased safety and security concerns.

As medicine moves beyond conventional clinical-based patient care to the personalized and preventative models offered by digital health applications, our concepts of patient safety will also have to change (30, 31). The current speed of technological advances also brings with it safety concerns. There is often a lack of quality and evidence-based research highlighting the associated health benefits of the newest technologies. There are also many challenges inherent in demonstrating that newer approaches and technologies, are indeed, effective (32).

The use of IoT devices in connected health means greater support for anywhere anytime solutions as well as real-time self-care or monitoring (33). However, transferring data from the point of collection, such as IoT devices, to remote serves, brings security and privacy concerns that need to be addressed (34). While the practices of informed consent and privacy by design are well-established in digital health, there are still concerns surrounding patients' understanding of how their data is being processed and by whom (35). An emerging trend, not just in digital health, but in health research in general, is increased patient engagement, treating the patient as a stakeholder in research, not just a data source (36). The perspective of patients is vital for gaining a real understanding of what security and privacy mean in the context of connected health (37).

At the time of writing, there is no universally established treatment or vaccine for COVID-19, the IoT can play a vital role as an information source and monitoring tool during the pandemic (38, 39) or potentially oncoming ones. Moreover, such devices can help in efforts to nowcast events, such as second waves via the harnessing of large amounts of physiological and behavioral data gathered from the general population (40). However, the collection of such data is not enough; complimentary artificial intelligence solutions are needed to exploit the saliency of such data (41).

## 5. Role of Artificial Intelligence

Artificial Intelligence can utilize data generated in digital health systems to help with aspects in medicine, such as improved diagnosis, selecting treatments, and predicting clinical outcomes. The presence of AI solutions in digital health intensifies challenges surrounding safety, explainability, and fairness (42). In regard to safety, AI systems are held to higher perceived safety standards than humans (43); i.e., it is less acceptable for AI to make errors. Moreover, the risk to human life of AI-based systems is, currently, not well-studied, and there is a lack of standards for the verification and validation of such systems. There are also generalization issues associated with AI models, reproducing promising results, made on “limited” training sets, on real-world data. A recent systematic review of deep learning solutions in medical images found that only a minimal number of studies in this field were of sufficient quality for clinical implementation (44).

With the recent introduction of the General Data Protection Regulation (GDPR) in the European Union, clinicians and patients have a right to understand how a particular AI decision was reached (45). The benefits of improving trust and transparency in AI systems will not only benefit clinicians and patients; increased knowledge and understanding into internal operations and decisions should also improve the overall accuracy and generalizability of the enhanced system (46). Therefore, for deployment in clinical settings, the next generation of AI technologies needs to be transparent, understandable, explainable, and fair.

Despite AI already having achieved remarkable results in a range of health-based detection tasks, increasing explainability is a highly non-trivial task. This difficulty arises as many of these results have been achieved using “black-box” techniques. That is, data is fed in, which in turn generates a predictive output, but the system does not provide any information or inference concerning how it arrived at the predicted value. This issue is particularly pronounced with the increasing presence of deep learning systems in digital health (47). Deep learning models have internal connections measuring in the millions (48).

## 6. The Potential of Genomics

Technological advances, increasing demand, and a reduction in costs mean that the amount of people undertaking genetic profiles is increasing. Despite this increase in supply and demand, outside of a handful of notable cases, such as rare disease diagnosis and cancer screening, genetic information is not integrated into routine medical care (49, 50). Genomics information has the potential to make considerable gains in data-driven, personalized care (51). For this potential to be met, there needs to be further developments in genetic risk scores relevant for broader clinical, and greater understandings into the interpretation of genetic variants (49).

These two factors are, of course, interlinked. Genomics, on one level, essentially provides information regarding “what might happen” to an individual concerning their health (52). A key factor in improving our understanding of this information is the interpretation of genetic variants identified during testing. There are many millions of these variants, and no standard definition for all of them (50, 53). Facilitating data and computational resource sharing are commonly identified means of closing this knowledge gap (50). Data sharing comes with increased ethical concerns, which have already been discussed in this article.

Genomics advancements are interlinked *connected health* challenges. For truly personalized medical care, genomics information should be combined with environmental, behavioral, and medical history information. Moreover, this combination of information from multiple heterogeneous sources needs to be performed in such a way that supports, not confounds clinicians and patients. These heterogeneous systems are highly dynamic and will most likely require advanced *artificial intelligence* paradigms to fully combine and analyse (49, 54).

Finally, genomics will play a vital role in efforts combating COVID-19, and any future similar pandemics. Increased genomic sequencing capabilities, accelerated through the use of AI, can help in the tracking of pathogens and viruses, and in the identification of their genomic signatures (55). Improved capabilities can also help identify genomic factors that increase an individual's susceptibility or resistance (56).

## 7. Conclusion

The current coronavirus disease 2019 (COVID-19) pandemic is undoubtedly challenging conventional medical services to their core. Digital health solutions are going to play a vital role in fighting the pandemic and potential future ones by enabling fundamental shifts in medical care both during the pandemic and in the aftermath. Such advancements are not going to come without challenges, both relating directly to the pandemic and more broadly to the development and implementation of new digital health solutions. Five years ago, *Multi-disciplinary Digital Health, Big Data and Public Health, MedTech, Self-Management, and Personalized Care, mHealth and Global Health Interventions, Evidence and Knowledge: Semantics, Social Media, and Persuasion, Serious Health Games and Games-Based Learning and Training*, and *Personal and Population Data—To Share or Not to Share?* were identified as some of the major challenges in digital health (9). These challenges are still very much at the forefront of digital health research. Rather than rehashing these challenges, this article has highlighted new concerns, with a focus on the role of digital health concerning COVID-19 and contagious virus diseases. By highlighting these challenges, Frontiers in Digital Health hopes to continue the trend of publishing world-class multi-disciplinary research addressing these and many other challenges in this exciting and constantly evolving field of research.

## Author Contributions

All authors listed have made a substantial, direct and intellectual contribution to the work, and approved it for publication.

## Conflict of Interest

The authors declare that the research was conducted in the absence of any commercial or financial relationships that could be construed as a potential conflict of interest.
